# Intensity simulation of photic effects after cataract surgery for off-axis light sources

**DOI:** 10.1371/journal.pone.0272705

**Published:** 2022-08-05

**Authors:** Pooria Omidi, Alan Cayless, Achim Langenbucher

**Affiliations:** 1 Department of Experimental Ophthalmology, Saarland University, Homburg, Saar, Germany; 2 School of Physical Sciences, The Open University, Milton Keynes, United Kingdom; University of Toronto, CANADA

## Abstract

Photopsia is a photic phenomenon that can be associated with intraocular lenses after cataract surgery. To calculate the relative light intensity of photic effects observed after cataract surgery at the foveal region as the most sensitive region of the retina, photopsia was simulated using the ZEMAX optical design software. The simulations are based on the Liou-Brennan eye model with a pupil diameter of 4.5 mm and incorporating implanted IOLs. The hydrophilic IOLs implanted in the eye model have a power of 21 diopter (D) with an optic diameter of 6 mm and 7 mm. Four different intensity detectors are located in specific regions of the eye in this simulation. The ray-tracing analysis was carried out for variations of incident ray angle of 0° to 90° (temporally) in steps of 1°. Depending on the range of incident ray angle, the light intensity was detected at detectors located on the fovea, nasal side of the retina, or the edge surface of the IOLs. Some portion of the input light was detected at specific incident angles in the foveal region. By altering the IOLs edge design to a fully reflective or anti-reflective surface, the range over which the light intensity is detected on the fovea can be shifted. Additionally, with the absorbing edge design, no intensity was detected at the foveal region for incident ray angles larger than 5°. Therefore an absorbing edge design can make photic effects less disturbing for patients.

## Introduction

Photopsia is an optical phenomenon that sometimes occurs shortly after cataract surgery. In this phenomenon light which is not directly correlated to an object is detected in the visual field. Clinical studies show that the photic effects caused by photopsia are mostly located in the temporal visual field. This effect has been named *dysphotopsia* by Tester et al. [1]. Masket et al. was the first to report that this effect could be associated with the optical design and position of IOLs in the eye [2] and Holladay et al. studied the effect of the IOL edge design on this phenomenon using a ZEMAX software simulation [3, 4].

Generally, patients do not suffer from photic effects after intraocular lens implantation. A few cases with these symptoms do not feel disturbed and they can tolerate this phenomenon [5, 6]. However, in rare cases, patients are severely disturbed by photopsia [3, 7, 8]. There is no clinical test to verify photopsia as this phenomenon cannot be measured objectively.

In the literature, several explanations for this phenomenon such as defects in the IOL optics during manufacturing, central optical defects during folding and injection [9], IOL material with a high index refractive index [5, 10, 11], optics with sharp or truncated edges [5, 10, 11], a cataract incision located temporally in the clear cornea [12], a prominent globe [13], lack of neural adaptation [14], and reflection of the anterior capsulotomy edge projected onto the nasal peripheral retina for photopsia, have been discussed. Moreover, there is no systematic correlation between photopsia and corneal diameter, anterior chamber depth, iris pigmentation, and photopic and scotopic pupil diameter or refraction [12].

In our previous study, we modelled the impact of the edge design of intraocular lenses (IOL) on the location, shape and relative intensity of photic effects at different regions of the retina [15].

In this paper, we have simulated the photic effects after cataract surgery in different regions of the pseudophakic eye. The main objective of this study is to calculate the relative light intensity of the photic effects on the foveal region as the most sensitive part of the retina, in order to better understand the effect of incident angles on the fixed eye model. Therefore we have defined our model based on parameters derived from the schematic model eye. Moreover, we have simulated the impact of the IOL edge design on the relative light intensity of the photic effects on the fovea.

## Methods

This study was registered at the local ethics committee of the Medical Association of Saarland (Ärztekammer des Saarlandes, 157/21).

### Eye model specifications

In this study, a pseudophakic eye model based on the Liou-Brennan schematic model eye introduced in 1997 was used for simulation [16]. Liou-Brennan model eye has an equivalent power of 60.35 D and an axial length of 23.95 mm.

The cornea of this model is defined by two rotationally symmetric surfaces (the front and back surface) with radii of 7.77 mm and 6.40 mm and asphericity of -0.18 and -0.60, respectively. The interspace between corneal surfaces is 0.5 mm with a refractive index of 1.376. The retina in defined by a half sphere with a radius of 12 mm and the fovea as the intersection of the visual axis and the retina with a diameter of 1.5 mm. The pupil diameter used in this model is 4.5 mm.

According to the schematic model eye of Liou & Brennan, all optical elements (corneal front and back surface, lens front and back surface) are centred on the ‘optical axis’. The pupil is somehow decentered by 0.5 mm in the nasal direction with respect to the optical axis to consider the incident ray angle of 5° from the nasal direction, as given by the model eye.

In this model, two acrylic hydrophilic IOLs with a refractive power of 21 D and refractive index of 1.458 are aligned with their equator in the equatorial plane of the crystalline lens of the eye model.

The front surface of the IOLs is defined by a spherical surface and the back surface by an aspherical surface optimized for 0.26 μm spherical aberration in the total eye model. These IOLs have a sharp circular optical edge with a thickness of 0.3 mm and optical diameters of 6 mm and 7 mm respectively. Finally, after IOL implantation in the model, the anterior chamber depth of the model eye is about 4 mm.

In our simulation we used these model eye data as a baseline and varied the incident ray angle to determine the critical ray incidence where (unwanted) light effects at the foveal region are observed. Table 1 lists all the relevant parameters of the proposed pseudophakic model eye and the polynomial coefficients that describe the rotationally symmetric aspheric surface of the IOL. Note that any polynomial coefficient values not specified in the table equal zero.

**Table 1:** Structural parameters of the pseudophakic eye model.

Surface	Medium	Radius[mm]	Asphericity	Thickness[mm]	Optical diameter[mm]
1	Cornea	7.77	-0.18	0.5	14
2	Aqueous	6.40	-0.6	3.16	14
3	Aqueous	∞	-	1	4.5
4	Hydrophilic IOL	13.86	-	0.97	6 and 7
5	Vitreous	-11.66	-1.5	18.32	6 and 7
6	-	-12	-	-	24
Surface	α_2_[mm^-1^]	α_4_[mm^-3^]	α_6_[mm^-5^]	α_8_[mm^-7^]	α_10_[mm^-7^]
5	-6.34e^-3^	1.15e^-3^	-3.86e^-7^	-2.47e^-8^	-

**Fig 1:**
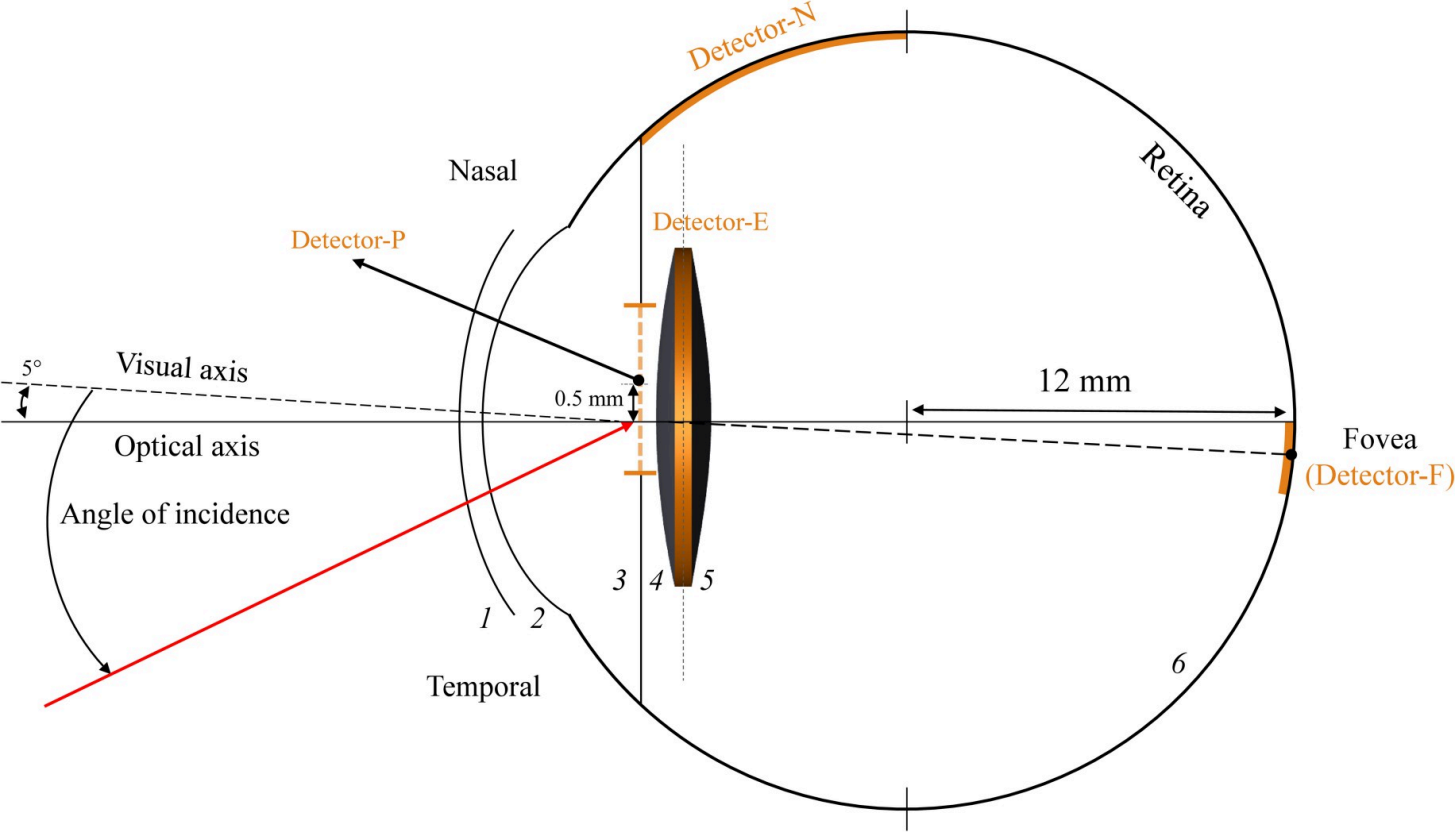
Schematic drawing of the pseudophakic eye and the detectors. (see Table 1 for parameter values).

To analyse the intensity of photic effects, four different light detectors are placed on different regions of the eye model. Fig 1 illustrates a schematic sketch of the eye model used in this study. Each surface is labeled with a number from 1 to 6 and detectors are shown in orange.

A circular light detector is located at the pupil to calculate the total intensity of light passing through the pupil. This detector is named as ’’Detector-P’’.

A second detector that named as ’’Detector-N’’ is located at the periphery of the nasal side of the retina and calculates the light intensity passing through the interspace between iris and IOL without hitting the lens.

Another detector is located at the edge surface of the IOLs and measures the intensity of the light hitting the lens edge surface. This detector is named ’’Detector-E’’.

Finally, the last detector named ’’Detector-F’’ is located at the foveal region to calculate the light intensity of the photic effects at the fovea.

Since the foveal region is the most sensitive region of the retina, even low intensities of light uncorrelated to the object space may cause optical disturbance for patients. The closer to the fovea, the more sensitive is the retinal surface.

To evaluate the contribution of the edge surface properties of the IOL on the intensity of the photic effects at the foveal region (Detector-F), three different edge designs with fully reflective, anti-reflective, and absorbing surfaces respectively were simulated.

With specular reflecting and anti-reflecting IOL edges, the effects of internal specular reflection and transmission are highlighted, respectively. By simulating an IOL with an absorbing edge, light transmission through and reflection off the edge is suppressed. These simulations provide insight to study the transmission, reflection, and absorption of the edge surface separately.

### Ray tracing procedure

Ray tracing was performed to calculate the intensity of photic effects reaching the foveal and retinal surface in comparison to the total power passing through the pupil. All of the simulations were carried out using the non-sequential ray-tracing mode of the ZEMAX professional ray-tracing software (Version 21.3, Washington, USA).

A collimated light pencil with 5 million rays from an extended light source is used to analyse the intensity of photic effects. The rays are distributed homogeneously over the pupil, and there is no weighting in their distribution function.

Rays from 0° to 90° degrees from the temporal direction in steps of 1° with respect to the visual axis (slanted by 5° to the optical axis) were traced temporally through the pupil to evaluate the intensity of the photic effects on the different detectors.

For each incident angle, the intensity obtained by the detectors are documented and referenced to the light intensity passing through the pupil.

## Results

**Fig 2:**
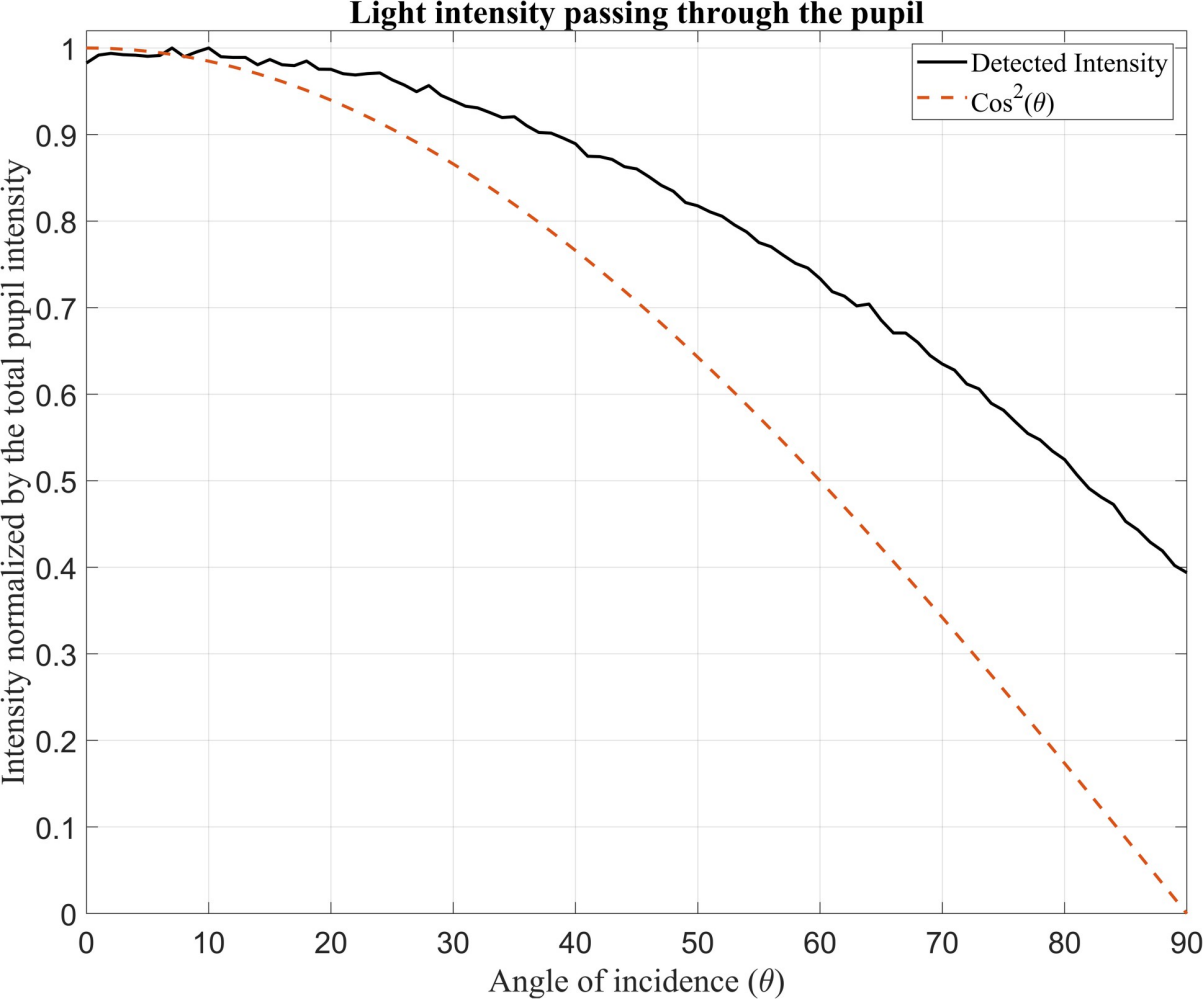
Normalised input intensity detected by the detector located on the pupil (Detector-P) over the incident ray angle.

Fig 2 shows the total input intensity detected by Detector-P as a function of the incident ray angle in comparison to the cosine squared function which resamples the intensity characteristics of a light pencil passing through a circular pupil.

With increasing incident ray angle, the total intensity of light decreases, but the difference between the normalised intensity of the light passing through the pupil and the cosine squared function increases.

**Fig 3:**
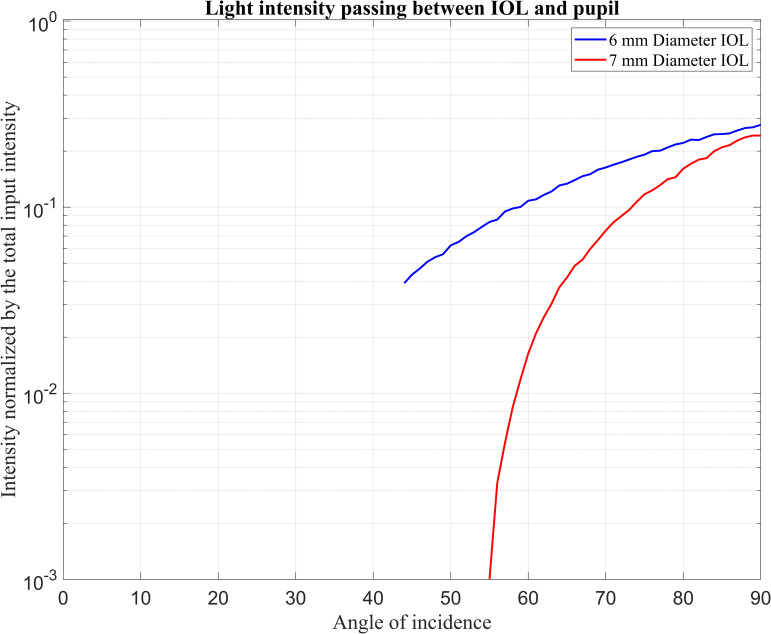
Normalised input intensity detected by the Detector-N over the incident ray angle.

Fig 3 shows the light intensity detected by Detector-N which passes through the pupil without interacting elements in between. Note that the light intensities are normalised to the total light intensity passing through the pupil at each angle.

It can be seen that rays passing through the interspace between IOL and pupil are detected for incident ray angles exceeding the critical angles 44° and 55° for IOLs with an optical diameter of 6 mm and 7 mm, respectively. The light intensity increases with the angle of incidence from these critical angles to 90°.

**Fig 4:**
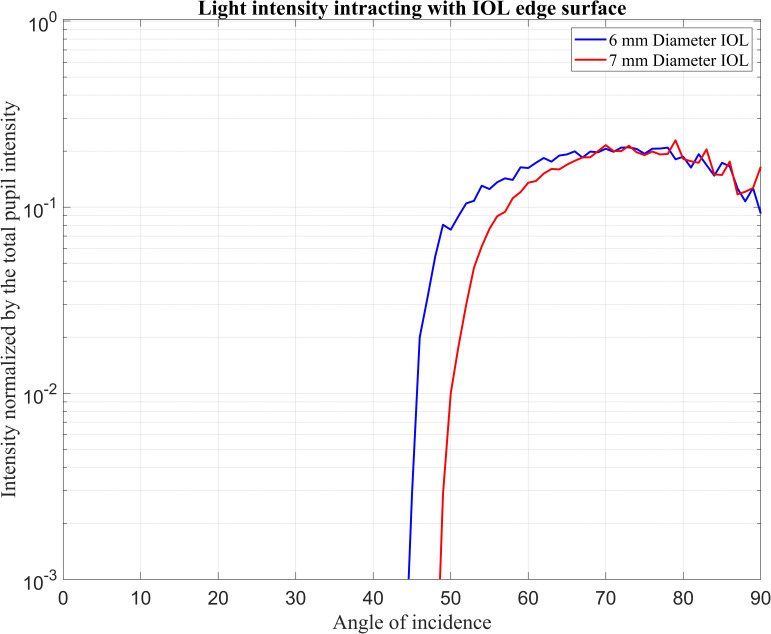
Normalised input intensity detected by the Detector-E over the incident ray angle.

Fig 4 shows the total intensity of the rays interacted with the IOL edges which are detected by Detector-E subdivided by the total intensity entering the eye (Detector-P) over the angle of incidence.

In this figure, rays from the incident angle of 42° start to interact with the edges of 6 mm IOL, and the rays from the incident of 48° start to interact with edges of 7 mm IOL. With increasing angle of incidence, the detected intensity at the edges initially increases dramatically, and then the intensity fluctuates slightly.

**Fig 5:**
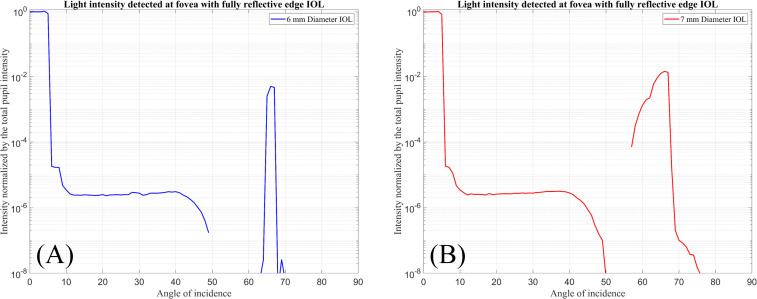
Normalised input intensity detected by Detector-F over the incident ray angle. (A) For 6 mm diameter IOL with fully reflective edge surface. (B) For 7 mm diameter IOL with fully reflective edge surface.

Fig 5 shows the total intensity of the rays hitting with the foveal (Detector-F) region normalised by the total intensity passing through the pupil when the IOLs are simulated with a fully reflective edge surface.

For small incident ray angles from 0° to 5°, almost all of the light passing through the pupil reaches the foveal region. For larger incident ray angles the light intensity on the foveal region decreases down to 0.001% of the light intensity passing through the pupil. In the range from 60° to 70°, about one percent of the total input light is detected at the foveal region.

**Fig 6:**
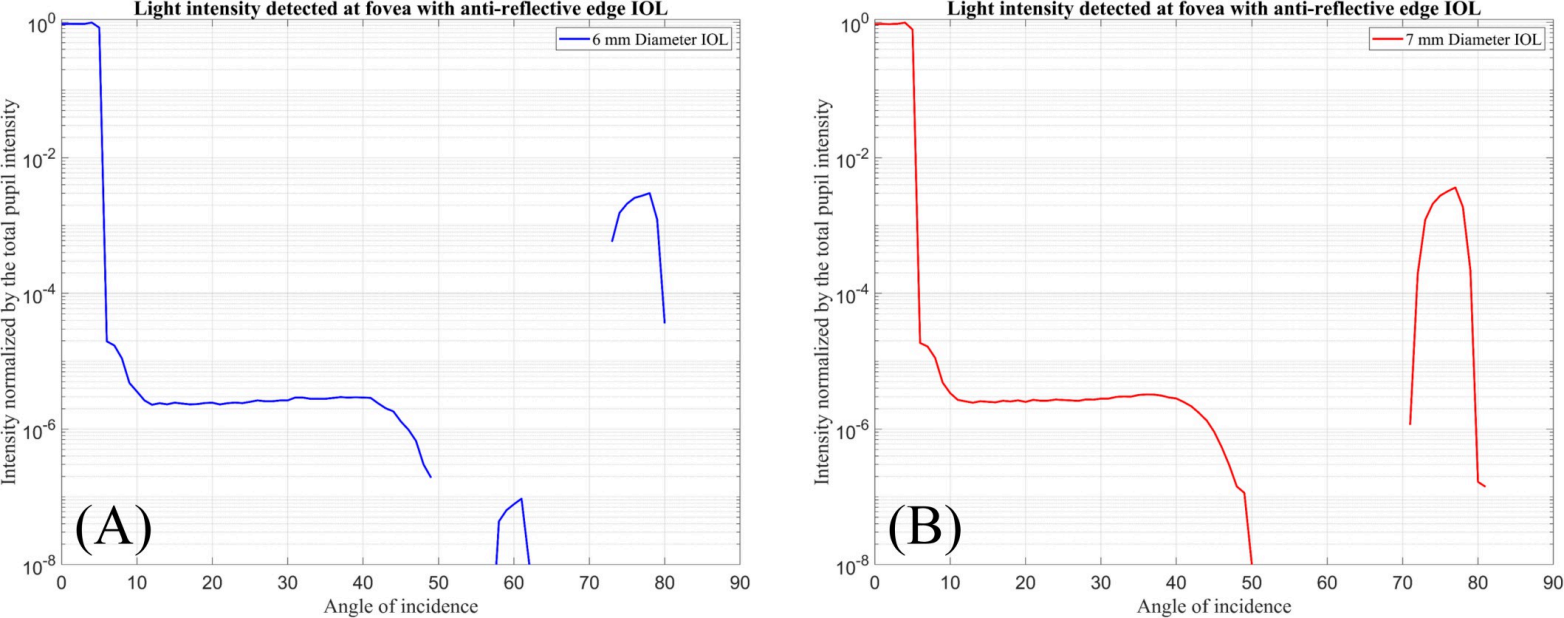
Normalised input intensity detected by Detector-F over the incident ray angle. (A) For 6 mm diameter IOL with anti-reflective edge surface. (B) For 7 mm diameter IOL with anti-reflective edge surface.

Fig 6 shows the total intensity of the rays hitting the foveal region (Detector-F) normalised by the total intensity passing through the pupil when the IOLs are simulated with anti-reflective edge surfaces.

In this figure, it can be observed that from incident ray angles between 70° to 80° about one percent of the light at the foveal region is detected. For the IOLs with 6 mm and 7 mm optical diameter, 0.3% of the input light is detected at angles of 77° to 78° respectively.

**Fig 7:**
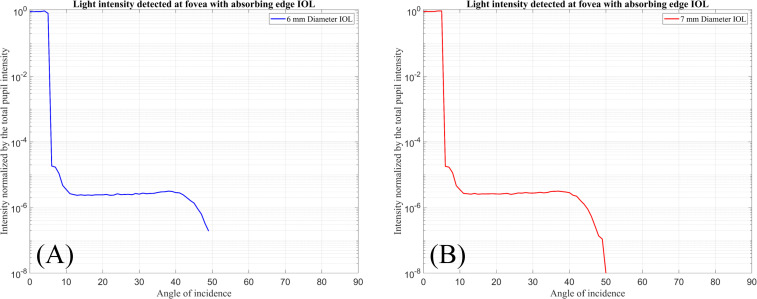
Normalised input intensity detected by Detector-F over the incident ray angle. (A) For 6 mm diameter IOL with absorbing edge surface. (B) For 7 mm diameter IOL with absorbing edge surface.

Finally, Fig 7 displays the total light intensity hitting the detector at the foveal region (Detector-F) normalised by the total light intensity passing through the pupil as a function of incident ray angle when the IOLs are simulated with absorbing edge surfaces.

In this figure, no intensity is detected at the fovea for incident ray angles exceeding 50°.

## Discussion

There have been several studies focused on IOL design and position to reduce photic effects [17]. Holladay et al. showed that the IOL edge design can affect the location and relative intensity of photic effects. Moreover, a sharp edge design of the IOL is one of the primary optical factors required for negative dysphotopsia [4]. Erie et al. modified the IOL design to reduce the intensity of photic effects. They showed that their new IOL design (having a peripheral concave posterior surface) provides more uniform illumination of the peripheral nasal retina and could thereby reduce negative dysphotopsia [18]. In the present study, we have modified the optical properties of the edge surface to investigate their impact on photic effects.

Standard ray-tracing techniques show that different incident angles cause the incoming light to be directed to various regions of the retinal surface. The intensity and location of these rays depend on how they interact with the IOL surfaces (front, back, edge). There is expected to be a correlation between the location of the photic effects and the amount of disturbance for the patients because different regions on the retina are differently sensitive to light.

In Fig 2 the black curve showing the total intensity passing through the pupil when a cornea is simulated in front is located above the red curve which shows total intensity passing through the pupil. As a result of the positive refracting power of the cornea, more light enters the eye at higher angles of incidence. These simulation results confirm that the illumination to the model eye has been implemented correctly.

In Figs 3 and 4, both detectors start to detect light intensity at lower angles for 6 mm IOL size. The standard explanation is that the 6 mm IOL is smaller than the 7 mm IOL and occupies less space in the eye. More space in the pseudophakic eye allows beams to reach the retina at lower angles. Moreover, in the 6 mm IOL, beams interacted with the edges of the IOL earlier (42°) than in the 7 mm IOL (48°). With increasing angle of incidence, the detected intensity at the edges initially increases dramatically, and then the intensity slope reduces. This phenomenon can be explained in terms of a range of incident ray angles covering the total edge surfaces.

In Fig 5, for the IOL with a 6 mm optical diameter, 0.5% of the input light is detected at an angle of 66° and for the IOL with a 7 mm optical diameter, 1.4% of the input light is detected at an angle of 67°. These intensities are caused by the reflection of beams entering the front surface and reflected from the IOL edge surface.

By changing the edge design from a fully reflective to an anti-reflective surface, the photic effects are detected within a different range of incident angles.

For incident ray angles in a range between 0° and 50°, the light intensities shown in Figs 5 and 6 are almost similar. These results show that with the specific incident ray angles light reflected or transmitted from the edges could cause disturbance for the patients.

Finally, Fig 7 shows that with an absorbing IOL edge, no intensity is detected at the fovea with incident ray angles exceeding 50°. Therefore, it can be concluded that all of the photic effects detected at the foveal region are generated by light reflected or transmitted from the edges. As a result, among these three IOL types, with absorbing edge IOL we can expect that patients will have less optical disturbance after cataract surgery.

Clinical studies in the future will be needed to prove the validity of our simulation model in a clinical environment.

### Limitations of this study

This study has the character of a pilot study and our principal aim has been to demonstrate the applicability of this concept. Our model in this study is based on the Liou-Brennan schematic model eye. This model reflects only an average geometry of a human eye. Since we would expect the behaviour of photic effects to vary across eyes with different proportions and corneal shape as well as pupil sizes/locations, it can be concluded that our results cannot be generalised to all human eyes on the basis of the current study alone.

## Supporting information

S1 Data (XLSX)
